# Comparison of MRI and digital breast tomosynthesis in the preoperative evaluation of multifocal breast cancer

**DOI:** 10.1186/bcr3773

**Published:** 2015-11-05

**Authors:** Bhavna Batohi, Valeria Vinci, Clare Peacock, Michael Michell, Asif Iqbal, David Evans, Juliet Morel, Keshthra Satchithananda, Reema Wasan, Rumana Rahim

**Affiliations:** 1 King's College Hospital, London, UK

## Introduction

Preoperative assessment of tumour extent is crucial in the management of breast cancer. MRI is currently indicated in cases of invasive lobular carcinoma on histology, a dense breast parenchymal pattern on 2D digital mammography (2DDM) or if there is a discrepancy between the clinical and radiological extent of disease. We compared the imaging characteristics of multifocal breast cancers on MRI, digital breast tomosynthesis (DBT), ultrasound and 2DDM to demonstrate the accuracy of each modality in the assessment of multifocal cancers.

## Methods

A retrospective review of 74 cases over a 4-year period was conducted. We included all cases whereby MRI or DBT identified two or more lesions that were considered suspicious or highly suggestive for malignancy. We compared the sign on MRI (including morphology and enhancement characteristics) against the lesion detectability on DBT. The final histology of these lesions obtained following ultrasound-guided core biopsy, vacuum-assisted MR-guided biopsy or surgical excision was considered.

## Results

There were 142 proven malignancies on histology out of the 74 cases, all of which were detected on MRI. The results of the MRI led to a change in surgical management in approximately 50% of cases but overstaged 16% of cases.

## Conclusion

MRI is more sensitive than the other three imaging modalities combined in accurately identifying multifocal breast cancer; however, DBT is still a useful adjunct in the evaluation of multifocal disease. There was no correlation between the pathological subtype and the non-detectability of multifocal cancer on the combined imaging modalities.

